# Practice patterns regarding regional corticosteroid treatment in noninfectious Uveitis: a survey study

**DOI:** 10.1186/s12348-021-00281-z

**Published:** 2022-01-04

**Authors:** Matthew McHarg, LeAnne Young, Natasha Kesav, Mehmet Yakin, H. Nida Sen, Shilpa Kodati

**Affiliations:** 1 National Eye Institute, National Institutes of Health, 10 Center Dr, 10/10N109, Bethesda, MD 20892 USA; 2 George Washington University School of Medicine, Washington, DC 20052 USA; 3 Cleveland Clinic Lerner College of Medicine, Cleveland, OH 44195 USA; 4 University Hospitals Eye Institute, Cleveland, OH 44106 USA

**Keywords:** Regional corticosteroids, Noninfectious uveitis, Practice patterns, Survey study, Pediatrics

## Abstract

**Background:**

Regional corticosteroid therapy for noninfectious uveitis is well-established but usage patterns have not been studied extensively. This study aims to assess practice patterns of retina and uveitis specialists regarding their preferences on the use of local corticosteroid therapy.

**Methods:**

A 13-question survey was developed regarding the practice patterns of regional corticosteroid use in specific situations and populations. The survey was distributed to both the American Uveitis Society and Macula Society.

**Results:**

Responses from 87 ophthalmologists were analyzed. The two most commonly used drugs were the dexamethasone intravitreal implant (Ozurdex®) and posterior sub-tenon’s triamcinolone (also known as posterior sub-Tenon’s Kenalog, or PSTK). Regional corticosteroids were used more frequently as first-line treatment in more than half of posterior uveitis cases when compared to anterior uveitis (39.1–46.0% vs 10.3%, respectively). Respondents were more willing to use regional corticosteroids in more than half of unilateral uveitis cases than in bilateral cases (54.7% vs 18.6%, respectively). A majority of respondents (67.1%) stated that they would avoid using regional corticosteroids in patients under 8 years old.

**Conclusions:**

Our results demonstrate more frequent regional corticosteroid use in posterior segment uveitis, unilateral cases, and avoidance in younger pediatric patients. Overall, the variability in these responses highlights the need for guidelines regarding regional corticosteroid use.

**Supplementary Information:**

The online version contains supplementary material available at 10.1186/s12348-021-00281-z.

## Background

The *uveitides* are a heterogenous group of inflammatory diseases and are a significant cause of blindness worldwide, accounting for up to approximately 5–15% of cases [1–4]. Uveitic diseases are caused by either infectious or noninfectious etiologies, with noninfectious cases comprising the majority of cases in developed countries [1, 5]. Uveitis can be anatomically classified into anterior, intermediate, posterior, and panuveitis depending on the predominant site of inflammation. Anterior uveitis is the most common worldwide, followed by posterior and panuveitis, with intermediate uveitis being the least common [5, 6].

Corticosteroids are the standard of care treatment for noninfectious uveitis and can be administered topically, regionally, or systemically [7]. Regional delivery via corticosteroid injections was developed to maximize drug delivery to the ocular target tissue over a defined period of time and minimize systemic adverse effects that result from oral formulations [8, 9] Regional corticosteroids are commonly used in the treatment of noninfectious uveitis, including in cases of active intraocular inflammation and uveitic macular edema [10–12]. Regional administration of corticosteroid is, however, associated with an increased risk of cataracts, ocular hypertension, glaucoma, and injection or implantation site related complications [11–13]. Despite these side effects, they provide several important benefits over systemic corticosteroids, including minimal systemic exposure, potentially longer-term local delivery, and improved patient adherence. Systemic corticosteroids are typically reserved for bilateral uveitis, systemic disease, or when topical/local therapies fail to control inflammation [14]. However, prolonged systemic therapy can result in numerous documented side effects including cardiovascular disease, osteoporosis, hyperglycemia, fat distribution, and general immunosuppression, among others [15].

A variety of regional corticosteroids are currently available for treatment of noninfectious uveitis. These include the preservative-free formulation of triamcinolone acetonide (Triesence, Alcon, Fort Worth, TX) administered intravitreally [16, 17] and the preservative-containing formulation of triamcinolone acetonide (Kenalog, Bristol-Meyers Squibb, New York, NY) used off-label for posterior sub-tenon’s injection [10, 11, 18, 19] . Other regional therapies include the bio-erodible dexamethasone intravitreal implant, Ozurdex (Allergan Inc., Irvine, CA) [20, 21], and the long-acting fluocinolone acetonide intravitreal implants such as Retisert (Bausch & Lomb, Rochester, New York, NY) [22, 23] and Yutiq (EyePoint Pharmaceuticals, Watertown, MA) [24, 25].

Several surveys pertaining to the use of immunomodulatory therapy in noninfectious uveitis [26, 27] have previously been published. However, only one of these assessed the use of corticosteroid therapy in adult uveitis, and was focused only on systemic corticosteroid therapy [28]. To the best of our knowledge, there are no existing survey studies that focus on regional corticosteroid usage in uveitis; therefore, our survey queried clinicians about their use of six commonly used regional corticosteroids in specific situations to gain a better understanding of practice patterns.

## Methods

### Survey

A 13-question survey was developed using SurveyMonkey (Supplemental Appendix 1). The survey was open from June 2019 through August 2019 and distributed to two groups of specialists described below. The first 5 questions surveyed demographics of each respondent (Supplemental Appendix 1, Q1 – Q5). Next, the survey probed respondents about their practice patterns when treating noninfectious uveitis in different anatomic locations (Supplemental Appendix 1, Q6, Q7), as well as the specific types of local corticosteroid therapies used as first-line treatment (Supplemental Appendix 1, Q7, Q8, Q12). For these questions, respondents had a choice of answers based on a four-point Likert scale (never, infrequently, half of cases, most of cases).

The remaining questions asked about age cutoffs for using local steroid injections in children (Supplemental Appendix 1, Q9), the use of local corticosteroids as maintenance therapy (Supplemental Appendix 1, Q10, Q11), and whether the results of a recent clinical trial (the POINT trial) [29] affected their clinical practice (Supplemental Appendix 1, Q13).

### Survey population

The American Uveitis Society is a highly selective group of uveitis specialists with 22.5% international membership. The Macula Society is another highly selective group with 28.5% international membership. Both societies require members to devote time to clinical care and/or research and to have several recent publications in peer-reviewed journals specific to immunology/inflammation or posterior segment disease, respectively. Links to the survey website were distributed through American Uveitis Society and Macula Society electronic mailing lists.

There is minimal overlap in their respective electronic mailing lists (24 individuals), and each respondent was only allowed to submit one survey response. Similar to the Esterberg et al. survey [27], these two groups were chosen as their members consist of uveitis and retina experts who are likely to have extensive experience with the use of regional corticosteroid therapy in noninfectious uveitis.

### Statistical analysis

Results are reported as percentages of complete responses per question. During analytical interpretation, “never” and “infrequently” responses were combined as “never/infrequently” for ease of interpretation in three questions (Supplemental Appendix 1, Q6, Q7, Q8). Similarly, “half of the cases” and “most cases” responses were combined as “more than half of cases” for the same three questions.

## Results

In total, 99 experts responded to the survey. Twelve of these respondents were excluded for filling out less than 85% of the survey, and a final number of 87 responses were used for analysis. No respondents submitted more than one survey. Sixty-one respondents (70.1%) indicated that they were fellowship trained in uveitis. Most respondents (59 respondents, 67.8%) received the survey through American Uveitis Society, while the remaining 28 respondents (32.2%) completed the survey via the Macula Society. Sixty-four of the respondents (73.6%) practiced in the United States, and 33 respondents (37.9%) had been practicing for more than 20 years. Respondent characteristics are summarized in Table 1.

**Table 1:** Respondent characteristics

	≥85% completion (*n* = 87)
**Electronic Mailing List**	
American Uveitis Society	59 (67.8%)
Macula Society	26 (29.9%)
Both	2 (2.3%)
**Fellowship training**	
Uveitis fellowship trained	61 (70.1%)
Not uveitis fellowship trained	26 (29.9%)
**Primary focus of practice**	
Medical Retina	20 (23.0%)
Surgical Retina	25 (28.7%)
Uveitis	39 (44.8%)
Other	3 (3.5%)
**Experience with treating uveitis**	
< 5 years	21 (24.1%)
6–10 years	14 (16.1%)
10–15 years	11 (12.6%)
16–20 years	8 (9.2%)
> 20 years	33 (37.9%)

### Commonly used regional corticosteroid therapies

The most commonly used therapies were the dexamethasone intravitreal implant (Ozurdex) (36.6%) and posterior sub-tenon’s triamcinolone (also known as posterior sub-Tenon’s Kenalog, or PSTK) (34.9%), followed by intravitreal triamcinolone acetonide (IVTA) (20.3%). Fluocinolone acetonide intravitreal implants (Yutiq and Retisert) were rarely used. When separated by focus of practice (medical retina, surgical retina, and uveitis), the two most commonly used therapies were similar between these groups. Medical retina specialists used PSTK at a rate of 40.5%, followed by Ozurdex at 32.4%. Surgical retina used Ozurdex most followed by PSTK, at rates of 38.8% and 32.7%, respectively. Similarly, uveitis specialists used Ozurdex (40.8%) most frequently followed by PSTK (35.5%). Furthermore, the rates of uveitis fellowship training did not impact which therapies were used, as both fellowship and non-fellowship trained specialists used Ozurdex and PSTK most commonly. Respondents who were uveitis fellowship trained commonly used Ozurdex at a rate of 39.2% and PSTK at 36.7%, while respondents without fellowship training commonly used both Ozurdex and PSTK at a rate of 33.3%.

### Use of regional corticosteroid therapy by anatomic location of uveitis

The data for different anatomic locations were divided into four subgroups: anterior uveitis, intermediate uveitis, posterior uveitis, and panuveitis. Nearly half of the respondents, 46.0%, used regional corticosteroids as first-line therapy in more than half of intermediate uveitis, while an average of 40.2% of respondents used it as first-line in more than half of posterior and panuveitis cases. First-line use of regional corticosteroids was less common in anterior uveitis, with only 10.3% using them in more than half of cases.

### Use of specific regional corticosteroid therapies

PSTK was the most frequently used first-line-therapy in intermediate uveitis with 31.8% using it first-line in more than half of cases. Ozurdex was used more frequently in posterior and panuveitis with 42.4% using it as first-line in more than half of cases. Lastly, results indicated that Yutiq and Retisert were rarely used in uveitis.

### Use of regional corticosteroid therapy for unilateral/bilateral cases and in the presence of systemic disease

A majority of respondents (54.7%) were willing to use regional corticosteroids as first-line therapy in more than half of unilateral uveitis cases, while only 18.6% were willing to use them in more than half of bilateral cases. Regional corticosteroids were also used infrequently in the presence of systemic immune-mediated disease, as only 24.7% used them in more than half of these cases.

### Use of regional corticosteroid therapy in children

Only 8.2% of respondents used regional corticosteroids as first-line therapy in children in more than half of cases. However, respondents were willing to use regional corticosteroids in children in certain cases. When asked about age cutoffs, 67.1% stated that they would avoid the use of regional corticosteroid therapy in children ≤8 years old. Avoidance of regional corticosteroids decreased with increasing patient age, with 41.5% avoidance for children ≤13 years old, and 9.8% avoidance in children ≤18 years old.

### Use of local corticosteroid therapy as maintenance therapy

Most respondents (72.1%) were willing to use local corticosteroid injections as maintenance therapy, defined as either repeated injections or long acting slow release systems such as Retisert or Yutiq.. Those who used local corticosteroids as maintenance therapy did so sparingly, with 82.3% of respondents using them in less than 25% of cases.

### Effects of POINT trial on practice patterns

Respondents completed the survey approximately one year after the POINT Trial results were released publicly. A majority of respondents (57.5%) reported their practice patterns did not change as a result of the POINT Trial.

## Discussion

There are multiple factors influencing the selection of local corticosteroid therapies in the treatment of noninfectious uveitis, including availability, cost, and provider comfort with specific therapies, among others. Additionally, co-management with rheumatologists and/or other specialists often impacts therapeutic considerations. While clinical efficacy is still regarded as one of the principal factors in determining therapy selection, the differences in efficacy can be overshadowed by the aforementioned external factors and may have affected survey responses. Furthermore, there are few guidelines specifying how these therapies should be implemented in different clinical scenarios, giving rise to more variability. Thus, the goal of this survey was to assess contemporary practice patterns of retina and uveitis specialists regarding their use of regional corticosteroids as a way to better understand indications and variations in practice.

In terms of specific therapies, PSTK was most commonly used in intermediate uveitis (38.8% of respondents used it in more than half of cases), while Ozurdex was most commonly used in posterior and panuveitis (42.4% of respondents used it in more than half of cases). The preference for PSTK and Ozurdex compared to IVTA may be due to the increased risk of intraocular hypertension with IVTA compared to PSTK [30, 31], although this was not demonstrated in the POINT Trial [29]. Additionally, given that sterile endophthalmitis has also been reported with intravitreal administration of triamcinolone acetonide [32, 33], PSTK can be considered preferential to IVTA in situations where Triesence® availability is limited. Lastly, separating these results by area of focus and fellowship training did not have a significant impact on the most commonly used therapies. This suggests that some consistency exists among all ophthalmologists regarding treatment of noninfectious uveitis.

Fluocinolone acetonide implants Yutiq and Retisert were used infrequently by respondents. The reasons for less frequent utilization of Yutiq can likely explained by both its high cost and the fact that it has only received approval from the US Food and Drug Administration (FDA) in 2018 [34]. Retisert, in contrast, requires invasive surgery and has significant side effects. For example, 90% and 45% of patients underwent cataract surgery and glaucoma surgery, respectively, in the 7-year Multicenter Uveitis Steroid Treatment (MUST) trial [35]. Therefore, the Retisert implant is typically reserved for cases that require long-term treatment. Finally, cost and access to Retisert may contribute to is low usage, as one respondent commented that Retisert was not available in their country.

As expected, our results showed that use of regional corticosteroids was less common in anterior uveitis, with 89.7% of respondents using them as first-line in less than half of cases. This finding is unsurprising, given that topical corticosteroid eye drops are typically used as first-line treatment of anterior uveitis [36]. In contrast, the use of regional corticosteroids was common in posterior segment uveitis, with 39.1–46.0% of respondents using it first-line in more than half of cases. In addition, the survey found that regional corticosteroids were rarely used as maintenance therapy – of the respondents who used regional corticosteroids as maintenance therapy, most (82.3%) did so in less than 25% of cases. This is likely due to concerns regarding the long-term cumulative effects of local corticosteroid treatment and the need for long-term immunomodulatory therapies in these patients.

Our results also show that clinicians were more likely to use regional corticosteroids in unilateral cases than bilateral uveitis (54.7% vs 18.6% in more than half of cases, respectively). This can be explained by the general understanding that unilateral cases constitute optimal candidates for regional corticosteroid therapy in the absence of obvious contraindications [37, 38].

There is limited data reporting the use of corticosteroids in pediatric populations. Carpentier et al. surveyed pediatric ophthalmologists and uveitis specialists on the management of uveitis-associated pediatric cataracts. They reported 64.5–71.0% of uveitis specialists used regional corticosteroids in perioperative management of cataracts compared to 29.8–68.1% of pediatric ophthalmologists [39]. Additionally, in the recent consensus report from the Paediatric Ocular Inflammation Group, no consensus was reached regarding the use of periocular steroids in juvenile idiopathic arthritis-associated uveitis [40]. Although there are undoubtedly unique considerations for the use of regional corticosteroid therapy in pediatric populations, especially with the concern for cataract formation [41], our survey found that there are situations where uveitis specialists would consider using them in children under the age of 18 (90.2% of specialists). Notably, our results show that avoidance of regional corticosteroid use in children decreased with patient age.

The POINT trial showed Ozurdex and IVTA to be superior to periocular steroid injections [29]. However, our survey did not show preferential use of Ozurdex compared to PSTK and the majority of respondents (57.5%) reported the recent POINT trial did not change their practice patterns. There may not have been adequate time to see practice pattern changes, as the publication of the POINT trial occurred only 4 months prior to distribution of the survey. Another explanation may be the lack of access to Ozurdex, as 26.4% of respondents were from outside the US and some practices or institutional pharmacies may not have it readily available in their formulary.

Strengths of the study included the prospective design and distribution to subspecialty societies with expertise in treating uveitis. The strict membership requirements of American Uveitis Society and Macula Society targeted the survey to a large group of ophthalmologists that were experienced in treating uveitis patients. Another strength is that, to the best of our knowledge, this is the first survey study assessing practice patterns of local corticosteroid usage in both pediatric and adult patients. Also, our sample of 87 respondents is of comparable size to similar electronic surveys/questionnaires in uveitis that have included between 45 and 132 ophthalmologist responses [27, 28, 42–45].

This study is limited by the sample size. Additionally, since there are a variety of factors that affect prescribing in various global healthcare environments and situations, it is difficult to generalize and fully capture the exact reasons behind therapeutic decisions regarding local corticosteroids. In his survey of uveitis specialists, Cunningham notably discusses that there has been a lack of consensus among uveitis specialists across a multitude of practice patterns, ranging from toxoplasmosis to noninfectious uveitis treatments [46]. He describes the need for consistently updated evidence-based treatment guidelines to ensure that both specialists and non-specialists can maximize positive patient outcomes. No such guidelines exist regarding corticosteroid use in noninfectious uveitis; nevertheless, our study does provide useful information and highlight these patterns of regional corticosteroid use in a variety of populations, anatomic locations of uveitis, and specific therapies. In summary, our results demonstrate a preference for the use of Ozurdex and PSTK in posterior segment uveitis, the use of regional corticosteroids in unilateral disease, and less frequent use as maintenance therapy. More research is required to accurately determine practice patterns for the use of regional corticosteroid therapies in the treatment of noninfectious uveitis. We hope that the reported experiences of highly experienced uveitis specialists in this survey can help to lay a foundation for more comprehensive comparative studies and guidelines in the future.

## Supplementary Information

**Additional file 1.** Supplemental Appendix 1: Survey regarding regional corticosteroid treatment in noninfectious uveitis.

## Data Availability

The datasets used and/or analysed during the current study are available from the corresponding author on reasonable request.

## Abbreviations

PSTK: posterior sub-tenon’s triamcinolone, also known as posterior sub-tenon’s Kenalog
IVTA: intravitreal triamcinolone acetonide
FDA: Food and Drug Administration
