# Supplementary material for: Practice patterns regarding regional corticosteroid treatment in noninfectious Uveitis: a survey study
Source: J Ophthalmic Inflamm Infect. 2022 Jan 4;12:3. doi: 10.1186/s12348-021-00281-z (PMC8727651; doi:10.1186/s12348-021-00281-z)
Supplement: Supplementary file 1 — Additional file 1. Supplemental Appendix 1: Survey regarding regional corticosteroid treatment in noninfectious uveitis. [file 12348_2021_281_MOESM1_ESM.docx]

# Supplemental Appendix 1: Survey regarding regional corticosteroid treatment in noninfectious uveitis.

1. ELECTRONIC CONSENT: You have read the above information. You voluntarily agree to participate. You are 18 years of age or older

- Agree
- Disagree

# Years of experience treating uveitis:

# <5 years

# 6-10 years

# 10-15 years

# 16-20 years

# >20 years

# Are you fellowship trained in uveitis?

# Yes

# No

1. Did you receive this questionnaire through the:
   - American Uveitis Society
   - Macula Society
   - Both

# Primary focus of your practice:

# Medical retina

# Surgical retina

# Uveitis

# Other

1. How often do you use regional corticosteroid injection therapy as first-line for the following cases of noninfectious uveitis? (Anterior Uveitis, Intermediate Uveitis, Posterior Uveitis, Panuveitis)

Anterior Uveitis

- Never
- Infrequently
- Half of cases
- Majority of cases
- N/A

Intermediate Uveitis

- Never
- Infrequently
- Half of cases
- Majority of cases
- N/A

Posterior Uveitis

- Never
- Infrequently
- Half of cases
- Majority of cases
- N/A

Panuveitis Uveitis

- Never
- Infrequently
- Half of cases
- Majority of cases
- N/A

# How often do you use each specific local corticosteroid therapy for treating noninfectious uveitis based on anatomic location?

# Dexamethasone Intravitreal Implant (Ozurdex) use in anterior uveitis

- Never
- Infrequently
- Half of cases
- Majority of cases
- N/A

# Dexamethasone Intravitreal Implant (Ozurdex) use in intermediate uveitis

- Never
- Infrequently
- Half of cases
- Majority of cases
- N/A

# Dexamethasone Intravitreal Implant (Ozurdex) use in posterior uveitis

- Never
- Infrequently
- Half of cases
- Majority of cases
- N/A

# Dexamethasone Intravitreal Implant (Ozurdex) use in panuveitis

- Never
- Infrequently
- Half of cases
- Majority of cases
- N/A

# Intravitreal Triamcinolone Acetonide (IVTA) use in anterior uveitis

- Never
- Infrequently
- Half of cases
- Majority of cases
- N/A

# Intravitreal Triamcinolone Acetonide (IVTA) use in intermediate uveitis

- Never
- Infrequently
- Half of cases
- Majority of cases
- N/A

# Intravitreal Triamcinolone Acetonide (IVTA) use in posterior uveitis

- Never
- Infrequently
- Half of cases
- Majority of cases
- N/A

# Intravitreal Triamcinolone Acetonide (IVTA) use in panuveitis

- Never
- Infrequently
- Half of cases
- Majority of cases
- N/A

# Posterior sub-Tenon’s Triamcinolone (also known as posterior sub-Tenon’s Kenalog, or PSTK) use in anterior uveitis

- Never
- Infrequently
- Half of cases
- Majority of cases
- N/A

# Posterior sub-Tenon’s Triamcinolone (also known as posterior sub-Tenon’s Kenalog, or PSTK) use in intermediate uveitis

- Never
- Infrequently
- Half of cases
- Majority of cases
- N/A

# Posterior sub-Tenon’s Triamcinolone (also known as posterior sub-Tenon’s Kenalog, or PSTK) use in posterior uveitis

- Never
- Infrequently
- Half of cases
- Majority of cases
- N/A

# Posterior sub-Tenon’s Triamcinolone (also known as posterior sub-Tenon’s Kenalog, or PSTK) use in panuveitis

- Never
- Infrequently
- Half of cases
- Majority of cases
- N/A

# Fluocinolone Acetonide Intravitreal Implant (Yutiq/Retisert) use in anterior uveitis

- Never
- Infrequently
- Half of cases
- Majority of cases
- N/A

# Fluocinolone Acetonide Intravitreal Implant (Yutiq/Retisert) use in intermediate uveitis

- Never
- Infrequently
- Half of cases
- Majority of cases
- N/A

# Fluocinolone Acetonide Intravitreal Implant (Yutiq/Retisert) in posterior uveitis

- Never
- Infrequently
- Half of cases
- Majority of cases
- N/A

# Fluocinolone Acetonide Intravitreal Implant (Yutiq/Retisert) use in panuveitis

- Never
- Infrequently
- Half of cases
- Majority of cases
- N/A

1. How often do you use regional corticosteroid injection therapy as first-line for the following cases of noninfectious uveitis (assuming no contraindication for local or systemic treatment)? (Bilateral Cases, Unilateral Cases, Presence of systemic immune-mediated disease, in children)

Bilateral cases

- Never
- Infrequently
- Half of cases
- Majority of cases
- N/A

Unilateral cases

- Never
- Infrequently
- Half of cases
- Majority of cases
- N/A

Presence of systemic immune-mediated disease

- Never
- Infrequently
- Half of cases
- Majority of cases
- N/A

In children

- Never
- Infrequently
- Half of cases
- Majority of cases
- N/A

# Under what age would you avoid using local injection, assuming patient has no contradictions to local or systemic treatment?

# ≤8 years old

# ≤13 years old

# ≤18 years old

# ≤30 years old

# Depends on patient

# No age limit

# N/A

1. Do you use local injection therapy as maintenance therapy (either repeated injections or long acting slow release systems such as fluocinolone acetonide (Retisert or Yutiq)?
   - Yes
   - No
2. If yes (to Question 10), how often do you use local therapy as maintenance?

- Less than 25% of cases
- 25-50% of cases
- Greater than 50% of cases

1. Which TWO do you most commonly use?

- Dexamethasone intravitreal implant (Ozurdex)
- Posterior sub-Tenon’s Triamcinolone (also known as posterior sub-Tenon’s Kenalog, or PSTK)
- Intravitreal Triamcinolone Acetonide (IVTA)
- Fluocinolone acetonide intravitreal implant (Yutiq)
- Fluocinolone acetonide intravitreal implant (Retisert)
- Other

1. Recently POINT trial results showed intravitreal triamcinolone acetonide and intravitreal dexamethasone implant (Ozurdex) were superior to periocular triamcinolone injections for the treatment of uveitic macular edema. Have the results of POINT Trial changed your practice?
   - Yes, I now prefer intravitreal steroids (Ozurdex or Triamcinolone) over periocular steroids
   - No, my practice did not change
